# An Apta-Biosensor for Colon Cancer Diagnostics

**DOI:** 10.3390/s150922291

**Published:** 2015-09-03

**Authors:** Mojgan Ahmadzadeh Raji, Ghasem Amoabediny, Parviz Tajik, Morteza Hosseini, Ebrahim Ghafar-Zadeh

**Affiliations:** 1 Department of Nanobiotechnology, School of New Sciences and Technologies, University of Tehran, Tehran14395-1561, Iran; E-Mails: m_ahmadzadeh@ut.ac.ir (M.A.R.); hosseini_m@ut.ac.ir (M.H.); 2 Department of Biotechnology and Pharmacy Engineering, Faculty of Chemical Engineering, University of Tehran, Tehran 4563-11155, Iran; 3 Research Center of New Technologies in Life Science Engineering, University of Tehran, Tehran 1417963891, Iran; 4 Department of Theriogenology, Faculty of Veterinary Medicine, University of Tehran, Tehran 1419963111, Iran; E-Mail: ptajik@ut.ac.ir; 5 Department of Electrical Engineering and Computer Science, York University, Toronto, ON M3J1P3, Canada

**Keywords:** aptasensor, aptamer, cyclic voltammetry (CV), colon cancer, biosensor, fluorescence labeling

## Abstract

This paper reports the design and implementation of an aptasensor using a modified KCHA10a aptamer. This aptasensor consists of a functionalized electrodes using various materials including 11-mercaptoandecanoic acid (11-MUA) and modified KCHA10a aptamer. The HCT 116, HT 29 and HEp-2 cell lines are used in this study to demonstrate the functionality of aptasensor for colon cancer detection purposes. Flow cytometry, fluorescence microscopy and electrochemical cyclic voltammetry are used to verify the binding between the target cells and aptamer. The limit of detection (LOD) of this aptasensor is equal to seven cancer cells. Based on the experimental results, the proposed sensor can be employed for point-of-care cancer disease diagnostics.

## 1. Introduction

**Figure 1:**
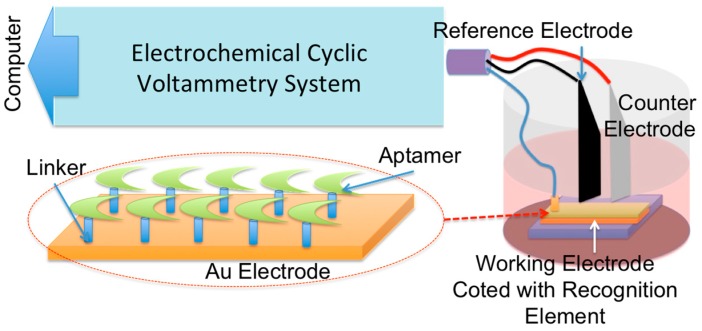
Illustration of an apta-sensor using the electrochemical reading technique.

Colorectal cancer is the second and third most common cause of cancer deaths in Canada and Iran, respectively [1,2]. Rapid diagnosis of this disease increases the chance of survival and decreases the medical management cost. Aptasensors have attracted attention for potential point-of-care diagnostic applications of a variety of deadly diseases such as prostate and colorectal cancers [3,4,5]. In these sensors, aptamers immobilized on the surface of electrodes play a key role as a recognition element for the detection of biomarkers associated with the various diseases. The interactions between aptamers and target cells/molecules are measured using various techniques, including optical and electrochemical ones [6,7,8]. The focus of this paper is on the design and implementation of an electrochemical aptasensor for colon cancer detection, as shown in Figure 1.

Since the late 1970s, carcinoembryonic antigen (CEA) has used as a major biomarker for the detection of colon cancer and other tumors of epithelial origin. CEA is a highly glycosylated biomarker which is expressed at the surface of the HCT 116 human cell line. This antigen provides selective high affinity binding to aptamers synthesized for colon cancer detection. Anti-CEA antibody can also be considered as an alternative for the detection of colon cancer [9,10,11]; however, in contrast to antibodies, aptamers offer the key advantages of greater specificity and affinity binding with target molecules. Indeed the small size of aptamers (<100 nucleotides [12]) in comparison to antibodies (~10 nm [13]), is the key factor in creating such high affinity and sensitivity. Further, aptamers are more stable under ambient conditions (e.g., temperature = 25°) than antibodies, and thus have a much longer shelf life. Additionally aptamers are produced through economical chemical process from large compound libraries containing different sequences using the systematic evolution of ligands by exponential enrichment (SELEX) procedure, while antibodies are routinely extracted from animals (e.g., rabbit, sheep *etc.*) [14,15,16,17,18]. Aptamers are self-refolding and reusable, while antibody-based biosensors are disposable. Based on the above mentioned reasons, high sensitivity and low cost aptasensors are best candidates for colon cancer screening [19].

The immobilization of aptamers on the surface of gold electrodes or gold nanoparticles [20,21] using various methods is an important step to develop a biosensor. A low complexity method for this purpose relies on the simple physical adsorption of DNA aptamers on the gold electrodes [22]. This method does not offer stable binding due to the relatively weak and unreliable van der Waals forces between the surfaces of electrodes and aptamers. On other hand, covalent chemical bonding techniques can be employed to develop stable and strong linkers. As described in the next section, self-assembled monolayers of substances such as 11-mercaptoundecanoic acid (11-MUA) are stacked with strong chemical binding to form a linker between the aptamers and working electrode [20,21,22,23].

In the remainder of this paper, we present the design and implementation of our functionalized sensor in Section 2. Thereafter, in Section 3, the materials and experimental protocols are described. In Section 4, we also demonstrate and discuss the experimental results followed by the conclusions in Section 5.

## 2. Aptasensor Design

In this section, we describe the experimental techniques for the development of an aptasensor dedicated to colon cancer detection. This section presents the synthesis of the aptamer and creation of recognition elements immobilized on the electrode surface.

### 2.1. Aptamer Synthesis

**Figure 2:**
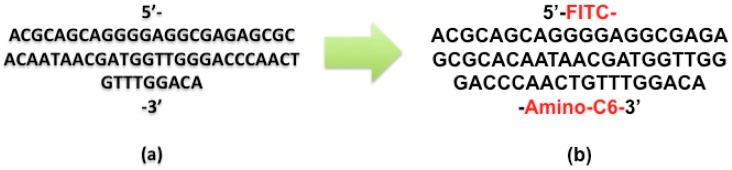
Synthesised DNA aptamer (**a**) prior to and (**b**) after labeling.

The aptamer synthesis is the first step toward the development of our biosensor. In this process, SELEX is employed to search for the aptamer DNA sequence. The following structure called KCHA10a (Figure 2a) is extracted through this process for colon cancer cell detection [19]. This aptamer sequence was functionalized by adding a linker and a label, namely amino-C6 at the 3′ end and fluorescein isothiocyanate (FITC) at the 5′ end [24]. This aptamer, called FITC-DNA Aptamer RAJI2-HP (Figure 2b), was synthesized by Tag Copenhagen Inc. (Frederiksberg, Denmark). 

**Figure 3:**
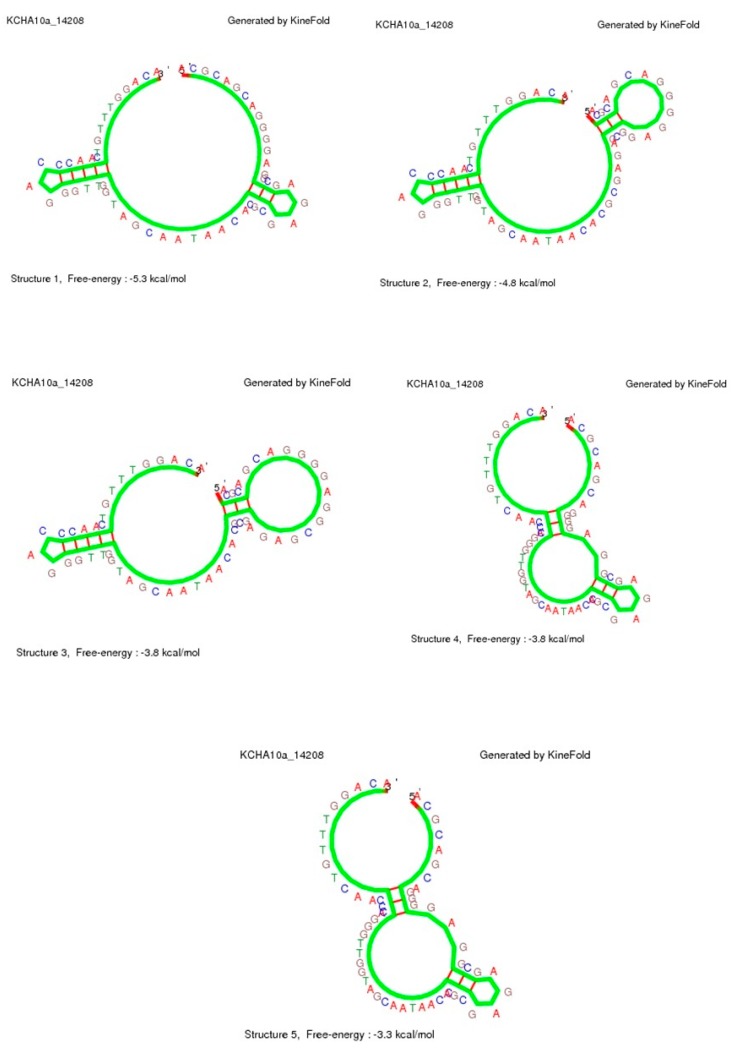
Simulation Results. Five predicated secondary structures of KCHA10a truncated aptamer.

The FITC label is used to detect the binding between the cells and aptamer using flow cytometry and fluorescence microscopy. Different secondary structures of this aptamer can be formed, as shown in Figure 3. These models are predicated using secondary structure prediction software [25]. As seen in this figure, five different structures are predicted for the secondary structure of the KCHA10a truncated aptamer. The lower free energy (E) in these predicted structures (−5.3 kcal·mol^−1^ < E <−3.3 kcal·mol^−1^) results in more stability in this nanosystem and consequently higher affinity to the colon cancer cells, confirmed by the 28.2 ± 2.7 nM value of Kd [12].

### 2.2. Recognition Element

A chemical procedure is performed to create a recognition element consisting of linkers connected to aptamers. In this paper, the following four-step procedure was employed to create the linker:

- Creation of thiol group by coating 11-MUA on electrode.
- Activation of COOH group in 11-MUA with ethyl(dimethylaminopropyl)carbodiimide (EDC)/*N*-hydroxysuccinimide (NHS).
- Binding with the NH_2_ at the 3′ end of the aptamer.
- Coating with bovine serum albumin (BSA) to prevent non-specific binding.

**Figure 4:**
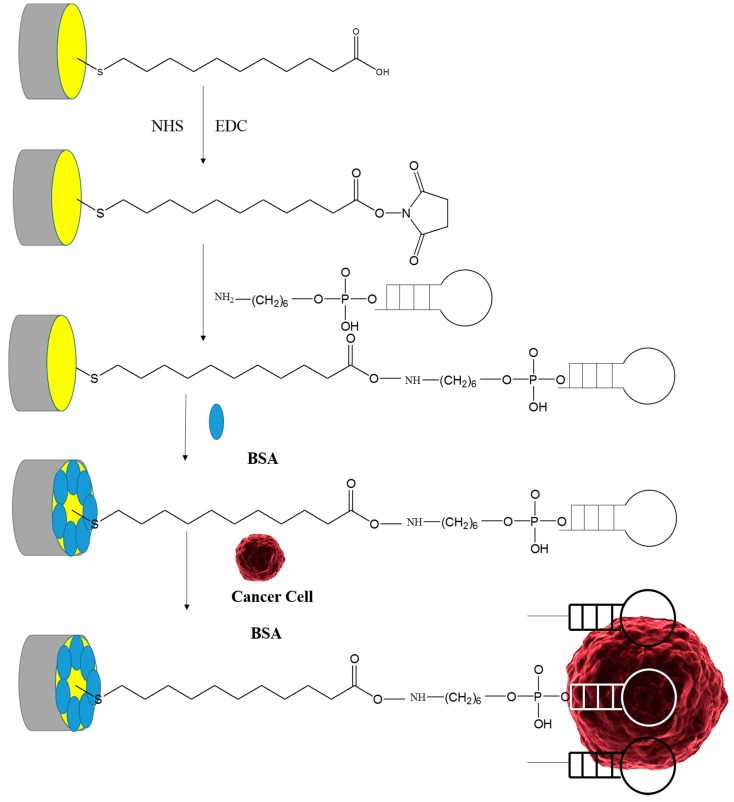
Schematic of the five step chemical process for the functionalization of an Au electrode dedicated for colon cancer detection.

Figure 4 shows all four steps of the creation of a single linker between the surface of an Au electrode (standard) and an aptamer molecule. However, the gold surface is covered with a large number of linkers terminated with aptamers for trapping and detecting the colon cancer cells.

## 3. Materials and Methods

### 3.1. Apta-Sensor Development

Gold electrode (Type: Au El., Code: 303013, Model: IRI.200-E, Azar Electrode Inc. (Orumieh, Iran) was cleaned prior to create the recognition element through four steps. In the first step, in order to coat thiol groups on the electrode surface, the electrode was soaked in ethanol/H_2_O solution 3:1 (v/v) containing 20 mM 11-MUA (95%, Sigma Aldrich, London, UK) for 18 h. Then the electrode was washed with ethanol and H_2_O, and dried with nitrogen gas. In the second step of this process, *N*-hydroxysuccinimide (NHS) and *N*-ethyl-*N*-(3-diethylaminopropyl)carbodiimide (EDC), also provided by Sigma-Aldrich, were used. EDC was used as a cross-linker to form an amide bond (Figure 4) and NHS was used to activate the carboxyl group associated with EDC. For this, the gold electrode was immersed in phosphate buffer saline (PBS) from Gibco^®^ (London, UK) for 1 h. This buffer, with an adjusted pH at 7.4, contained 2 mM EDC and 5 mM NHS. Thereafter the electrode was soaked in Tris-HCl buffer for 2 h. This buffer with an adjusted pH at 7.6 and adjusted ionic strength I at 0.14 contained 0.4 µM aptamer. In the last step, in order to avoid non-specific binding, electrodes were dipped in distilled water containing 1% BSA for half an hour. They were also soaked in Tris-HCl buffer [20,26] for 10 min to remove the unbonded BSA.

### 3.2. Cell Culture

In this project, three different cells were employed to verify the functionality of the apta-sensor. The device was tested using two cells associated with human colon cancer namely epithelial cancer cell lines (Pasteur Institute of Iran, Tehran, Iran); HCT116 (NCBI code: C570) and epithelial-like cancer cell line HT 29 (NCBI code: C466). Epithelial cell HEp-2 was also used as a control cell line. Also, McCoy’s 5A modified medium (Pasteur Institute of Iran, Catalogue number: 30-2007 from Gibco) was used to culture the colon cancer cells. This medium contained l-glutamine, penicillin, streptomycin, amphotericin B at concentrations of 300 mg/L, 100 μg/mL, 100 IU/mL, and 2.5 μg/mL, respectively. Also it contained fetal bovine serum, epidermal growth and adhesion factors (Nano Zist Arrayeh Inc., Tehran, Iran) along with antitrypsin activity factor to promote cell proliferation and cell attachment in the adherent flasks provided from JET BIOFII. For the control cells, RPMI (Bioeideh Inc., Tehran, Iran) was used as cell culture medium. To culture all types of cells, the incubation was performed in a SANYO device (model MCO-17AI, LabX, Tokyo, Japan) at 37 °C and carbon dioxide concentration was set to 5%. When control HEp-2 cells are confluent (25 cm^2^ flasks, JET BIOFIL, Guangzhou, China) the passage of cells was performed by discarding the culture medium and trypsinizing the cells using Ethylenediaminetetraacetic acid (EDTA) solution containing 0.25% w/v trypsin plus 0.53 mM EDTA [27]. However, for other cell lines, a non-enzymatic solution or cell scraper should be used instead of trypsin when the aptamer is attached to cells, because CEA is expressed on the cell surface and trypsin damages the cells. Detached cells were washed with washing buffer, centrifuged at 1200 RPM for 5 min, re-suspended in binding buffer and kept in room temperature for 20 min. An inverted microscope (IX70, Olympus, Tokyo, Japan) was used for the assessment and control of cell culture.

Two binding and washing buffers were also used to enable the conjugation of aptamers to cells with high affinity and sensitivity. Washing buffer containing 5 mM MgCl_2_ and 4.5 g/L glucose were used for rinsing the cells after exposing the cells on the aptasensor. The binding buffer contained 1 mg/mL BSA in washing buffer [28].

### 3.3. Flow Cytometry & Fluorescent Microscopy

A flow cytometer (Partec, Nuremberg, Germany) and fluorescence microscope (BX50, Olympus) were employed for the assessment of binding between the aptamer and cells. For this purpose, FITC was applied as a fluorescein molecule functionalized with an isothiocyanate reactive group (–N=C=S), with excitation and emission spectrum peak wavelengths ranging from 495 to 519 nm. This spectrum range was detectable in the FL1 channel of the flow cytometer after gating and determining the desired range of negative control cells. Furthermore, as the positive and negative control of these experiments, aptamers with 400 nanomolar and zero concentrations were used, respectively.

### 3.4. Electrochemical Experiments

All electrochemical measurements were performed at 25 °C temperature in PBS buffer containing K_3_[Fe(CN)_6_] with 1 mM concentration [20]. PBS adjusted at a pH equal to 7.4 was used as an electrolyte in this measurement procedure. Cyclic voltammetry (CV) was used as an electrochemical technique to detect the materials coated on the gold electrode layer by layer. These materials include 11-MUA, EDC/NHS and aptamer.

## 4. Results

### 4.1. Fluorescence Microscopy

**Figure 5:**
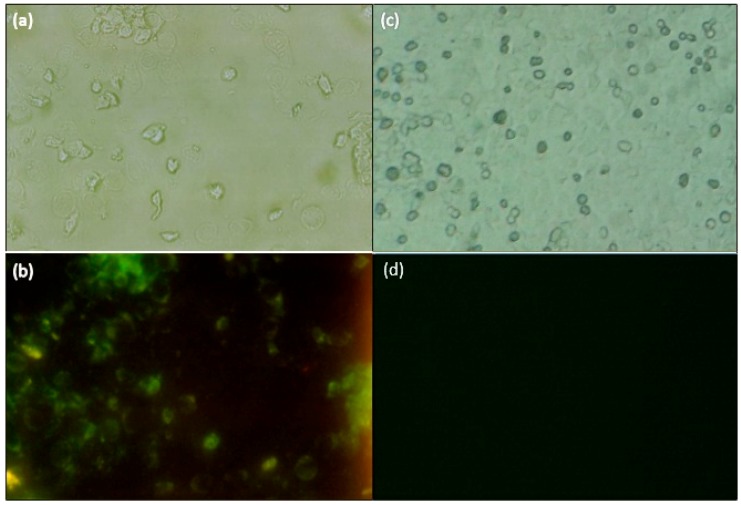
Fluorescence microscopy results: HCT 116 cell lines (**a**) prior and (**b**) after UV light; HEp-2 cell line (**c**) before and (**d**) after UV exposure (Olympus Fluorescence Microscope Model BX50, 40× magnification, Tokyo, Japan).

The binding of colon cancer cells to the aptamer were studied by fluorescence microscopy using the HCT 116 and HEp-2 cell lines. Figure 5a–d shows the microscopic images of these cells prior (Figure 5a,c) and after UV light exposure (Figure 5b,d). Based on these results, the binding between the aptamer and HCT 116 cells was confirmed (Figure 5b), while no binding between the HEp-2 cells and aptamer were indicated (Figure 5d). These experiments were repeated for three times. 

### 4.2. Flow Cytometry

**Figure 6:**
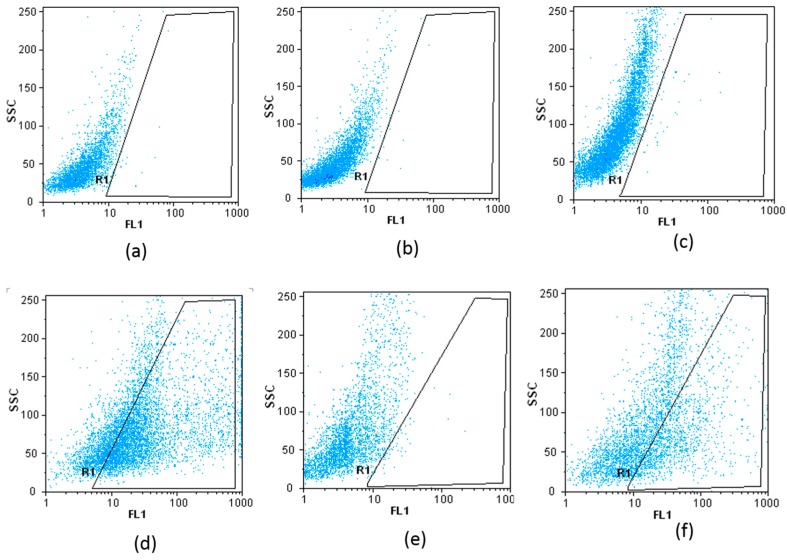
Flow cytometry histograms. (**a**,**b**) Epithelial cell line (HEp-2); (**c**,**d**) Epithelial cell line HCT 116 and (**e**,**f**) Epithelial-like cell line HT 29, before and after binding with aptamer are demonstrated. Experiments were repeated three times.

Flow cytometry is the best technique to verify the binding between the target cells and aptamer. In this experiment, the binding between HCT 116 and HT 29 cells and aptamers was studied. As already mentioned, HEp-2 cells are used as negative control. Figure 6a–f shows the side scatter cytometry (SSC) or granularity of different types of cells in the presence and absence of aptamers as a function of the cell numbers measured by the first channel of flow cytometer (FL1). For instance, Figure 6a,b indicates that there was no difference between the surface’s complexity of HEp-2 cells, before and after binding with aptamers. The significant difference between the two graphs (Figure 6c,d) reveals the interaction between the HCT 116 and aptamers. Less interaction between the HT 29 cells and aptamer is expected, as seen in Figure 6e,f. Flow cytometry histograms confirmed 60.19% and 29.62% aptamer connection to HCT 116 and HT 29 respectively. The percentage of attached cells analysed with the Flomax software is also shown in Table 1. Based on these results, the R1 region indicates the interaction with cells before and after introducing aptamers.

**Table 1:** Results of binding between HEp-2, HCT 116 and HT 29 and modified KCHA10a aptamer.

	%Gated (R1 Region)	Without Aptamer	Aptamer Attached
Cell Line			
HEp-2		0.2	0.2
HCT 116		0.43	60.19
HT 29		0.40	29.62

### 4.3. Electrochemical Experiments

#### 4.3.1. Functionalization of Electrodes

**Figure 7:**
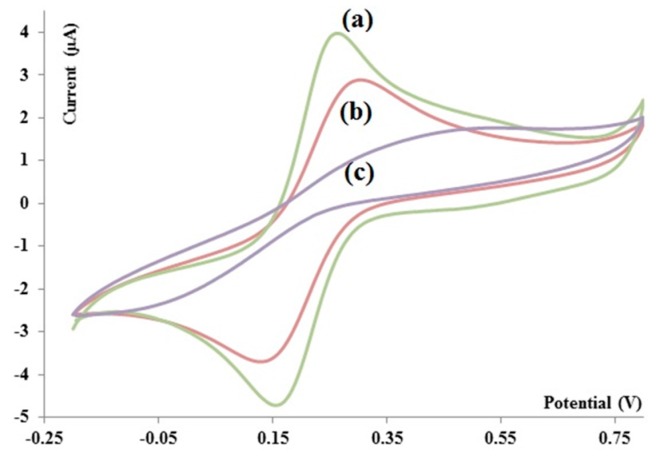
Voltammogram of bare (**a**) Au; (**b**) Au coated with 11MUA/EDC/NHS and (**c**) Au coated with 11MUA/EDC/NHS/APT/BSA in K_3_[Fe(CN)_6_] 1 mM in PBS (pH 7.4) *vs.* Ag/AgCl.

Electrochemical techniques such as cyclic voltammetry offer a great tool to study the self-assembled monolayer of different materials involved in creating linker. In Figure 7, the voltammograms of bare Au electrode, the electrode coated with 11 MUA/EDC/NHS, and the functionalized electrode in the presence of aptamer after BSA treatment, as shown with various colors—green, red and purple—respectively. In these experiments, Ag/AgCl is used as reference electrode. The scan rate was 50 mV/s in the range of −0.2 to 0.8 V. Figure 7 verifies that the amount of current is decreased after every coating step. In other words, the functionalization of the electrode is verified by these voltammograms.

#### 4.3.2. Elctrochemical Biosensor

**Figure 8:**
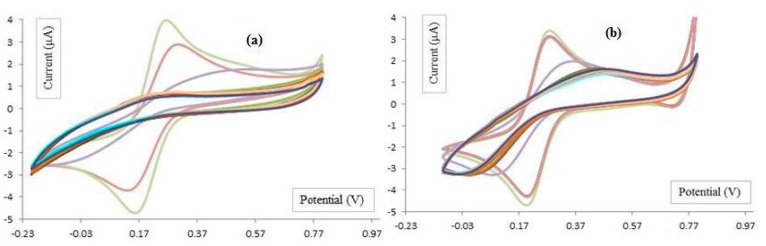
Electrochemical CV results, Au electrode, Au electrode coated with 11-MUA, Au electrode coated with 11-MUA terminated with aptamer and the functionalized electrode conjugated with different concentrations (6, 12, 100, 1000 cell/mL) of (**a**) HCT116 cells; (**b**) HEp-2 cells. Electrolyte was K_3_[Fe(CN)_6_] 1 mM in PBS (pH 7.4) *vs.* Ag/AgCl.

The CV results of apta-sensor in the presence of different concentrations of HCT 116 (6, 12, 25, 50, 100, 1000, 17,000 cells/mL) is showed in Figure 8a. Also the effect of different concentrations of HEp-2 cell line as a control cell (6, 12, 100, 1000 cells/mL) on apta-sensor is shown in Figure 8b. As described in Section 5, the CV results confirm the high affinity of colon cancer with aptamer in contrast with the control cells exposed to apta-sensor. 

## 5. Discussion

### 5.1. Self-Assembly of Monolayers on Electrodes 

These experiments performed on the electrodes coated with 11MUA/EDC/NHS and 11MUA/EDC/NHS/APT/BSA. In other words, as the negative charge density on the surface of electrode was increased, the current of Fe (CN)_6_^3−^ was decreased accordingly. Therefore, the results shown in Figure 6 demonstrate assembly of monolayers on the surface of Au electrodes.

### 5.2. Colon Cancer Cell Detection

**Figure 9:**
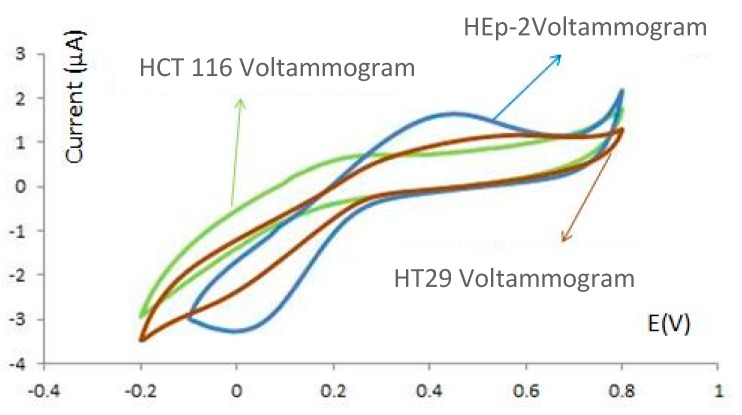
The voltammogram of HEp-2, HT29 and HCT 116 attached to sensor surface in K_3_[Fe(CN)_6_] 1 mM in PBS (pH 7.4) *vs.* Ag/AgCl.

As shown in Figure 8, the current peak in the presence of cancer and control cells is lower and higher than 1 µA, respectively. This current change may be caused by the higher affinity of cancer cells to conjugate with aptamer, while there is no affinity between the control cells and aptamer. As shown in Figure 8, with the higher concentration of target cells, the higher conjunction between the cells and functionalized electrodes occurs. Consequently, the peak current in each step is decreased. Let us assume the surface of the bare electrode is A_0_. It is expected that reduction and oxidation of Fe(CN)_6_ ions occurs during the CV experiment. When 11-MUA is deposited on the electrode, the surface is equal to A_1_. The surface is changed to A_2_ after covalent binding of aptamer and linker. Depending on the concentration of cells, the adherent of cells to the surface of electrode changes the surface to A_3_. As the results of the change of surface area (A_0_ > A_1_ > A_2_ > A_3_), the current peak at analyte oxidation (Ipa) and reduction (Ipc) changes as well. In the other words, Ipa_3_ < Ipa_2_ <Ipa_1_ < Ipa_0_ and Ipc_0_ < Ipc_1_ < Ipc_2_< Ipc_3_. The decrease in current oxidation peak (Ipa) and reduction (Ipc), exhibited lower oxidation and reduction of ions because of the changing charge density on the surface of each electrode. The CV results associated with colon cancer models and control cells with different concentrations can be extracted from Figure 8 and shown in Figure 9. This figure shows the difference between the voltammograms of the electrochemical sensor in the presence of HEp2, HT29 and HCT 116 cells.

### 5.3. Sensitivity of the Aptasensor

**Figure 10:**
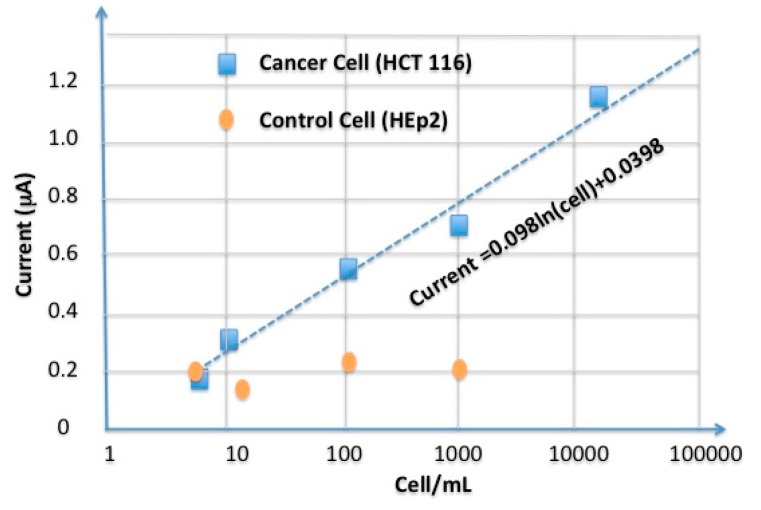
Calibration curve for cancer cells (HCT 116) and control cell (HEp-2).

The current peaks as a function of voltage for different cell types and cell concentrations are depicted in Figure 10. Based on the results, the saturation threshold of this sensor is 100 cells. When the number of cells exceeds this amount, the sensor loses its sensitivity. Based on the exploration shown in Figure 10, the sensor demonstrates a linear relation between the output current in the range of 1 to 100 cells. Theoretically, at the level of a single cell, the output current of the sensor results in a 0.03 µA change. Also, based on this discussion, the noise level can reach 0.2166 µA and the sensor might be capable of detecting more than seven cells (>0.2166/0.03~7).

### 5.4. Selectivity of the Aptasensor

A comparison between the results of the cancer and control cells illustrates that the sensor function is totally selective for the cancer model cells (Figure 10). The control cells generate a static output because these cells does not increase the output current of sensor. In the case of the cancer cells, an increase in the number of cells leads to an increase of the sensor’s output current.

## 6. Conclusions

This paper reports the design and implementation of an apta-sensor for colon cancer detection. We have demonstrated and discussed the functionality and applicability of the synthesized KCHA10a aptamer using flow cytometry, fluorescence microscopy and electrochemical experiments. The HCT 116, HT 29 and HEp-2 cell lines were used as colon cancer model and control cells, respectively. The surface of an Au electrode was coated—with SH groups using 11-MUA, EDC/NHS and aptamer. Furthermore, we put forward an apta-sensor demonstrating a linear relation between the numbers of cells (<100) with seven cell resolution. Based on the experimental results and discussions in this paper, the proposed apta-sensor offers high sensitivity and can be a good candidate for colon cancer diagnostics.
